# Dual kinetic curves in reversible electrochemical systems

**DOI:** 10.1371/journal.pone.0173786

**Published:** 2017-03-30

**Authors:** Michael J. Hankins, Gregory S. Yablonsky, István Z. Kiss

**Affiliations:** 1 Department of Chemistry, Saint Louis University, Laclede Av., St. Louis, MO, United States of America; 2 Parks College of Engineering, Aviation and Technology, Saint Louis University, Lindell Blvd, St. Louis, MO, United States of America; Universiteit Gent, BELGIUM

## Abstract

We introduce dual kinetic chronoamperometry, in which reciprocal relations are established between the kinetic curves of electrochemical reactions that start from symmetrical initial conditions. We have performed numerical and experimental studies in which the kinetic curves of the electron-transfer processes are analyzed for a reversible first order reaction. Experimental tests were done with the ferrocyanide/ferricyanide system in which the concentrations of each component could be measured separately using the platinum disk/gold ring electrode. It is shown that the proper ratio of the transient kinetic curves obtained from cathodic and anodic mass transfer limited regions give thermodynamic time invariances related to the reaction quotient of the bulk concentrations. Therefore, thermodynamic time invariances can be observed at any time using the dual kinetic curves for reversible reactions. The technique provides a unique possibility to extract the non-steady state trajectory starting from one initial condition based only on the equilibrium constant and the trajectory which starts from the symmetrical initial condition. The results could impact battery technology by predicting the concentrations and currents of the underlying non-steady state processes in a wide domain from thermodynamic principles and limited kinetic information.

## Introduction

Onsager relationships [1–3] are widely used for extracting of detailed information about reciprocal processes. Using these relationships one can measure (see, for example, reference [4]) how process I affects process II and extract the reciprocal information, how process II affects process I. These reciprocal relations have been verified experimentally [5], in particular for irreversible processes (thermoelectricity, electrokinetics, isothermal diffusion etc.) in physical systems. However, experimental studies regarding the Onsager relationships with chemical reactions are challenging. [6] Within the Onsager’s approach, the fluxes in the near-equilibrium-domain are linear functions of chemical potentials, and the reciprocal relations state that the coefficient matrix of these functions is symmetric. For chemical systems, it is impossible to measure these coefficients directly, as one has to solve the inverse, often ill-posed problem of chemical kinetics.

Nonetheless, it was demonstrated [6] that the reciprocal relations strongly impact the kinetic curves of reversible chemical reaction systems. Instead of differentiating the curves for rates of chemical reactions, the concentration vs. time curves can be directly compared for different, symmetrically related initial conditions. These dual kinetic experiments represent an alternate means for testing the Onsager reciprocal relations.

Mathematically, the relations between kinetic curves use the symmetry of the propagator in the special entropic inner product. [6] For linearized kinetics with the microreversibility, *dc/dt* = *Kc*, where *c* is a vector of concentrations, the kinetic operator *K* is symmetric in the entropic inner product. This form of Onsager’s reciprocal relationship implies that the shift in time, exp(*Kt*), is also a the symmetric operator. This generates reciprocity relations between concentration curves which can be measured experimentally [6]. A dual experiment can be defined for each ideal kinetic experiment. For this dual experiment, both the initial data and the observables are different (they exchange their positions), but the ratio of the corresponding concentrations is time invariant quantity.

Such virtual experiments have been proposed for sets of monomolecular reactions [7] and single nonlinear reaction [8]. The results of the dual kinetic experiments are the existence of time invariant quantities expressed as ‘equilibrium relationships for non-equilibrium dependencies’. The simplest example of such invariants can be presented using the reversible first order reaction

A⇄B (1)

where *A* and *B* are reactant and product chemical species respectively. Using this equation, the rate of change in concentration for each species can be written as:

$$\frac{dc_{A}}{dt}=-k_{f}c_{A}+k_{r}c_{B}$$ (2)

$$\frac{dc_{B}}{dt}=k_{f}c_{A}-k_{r}c_{B}$$ (3)

where *c*_*A*_ and *c*_*B*_ are concentrations of *A* and *B*, and *k*_*f*_ and *k*_*r*_ are the forward and reverse first-order rate constants. The equilibrium constant, *K*, is defined by:

$$K=\frac{k_{f}}{k_{r}}$$ (4)

When the time-dependent concentration is studied at symmetrical far-from-equilibrium conditions, the reciprocal behavior of the system is revealed. In the first experiment, starting from the initial condition only species *A* present (e.g. *c*_*A*_ = 1 M and *c*_*B*_ = 0). The concentration of *B* is:

$$c_{B}(t)=\frac{k_{f}[1-\exp\left(-k_{f}+k_{r}\right)t]}{k_{f}+k_{r}}$$ (5)

Likewise, in the second experiment starting from the symmetric second condition in which only species *B* is present (i.e. *c*_*A*_ = 0 and *c*_*B*_ = 1 M), the concentration of *A* can be presented as:

$$c_{A}(t)=\frac{k_{r}[1-\exp\left(-k_{f}+k_{r}\right)t]}{k_{f}+k_{r}}$$ (6)

Obviously, the ratio of concentrations *c*_*B*_*(t)/c*_*A*_*(t)* is an invariant quantity (*K*) for the entire reversible reaction:

$$\frac{c_{B}(t)}{c_{A}(t)}=K\;\text{for any}\;t>0$$ (7)

It was shown that such invariant relationship is not simply a consequence of the relatively simple kinetics: it is a general property of linear systems (due the symmetry of the time shift operator in the entropic inner product) and one single non-linear reaction as well [6]. This theoretical fact provokes a formulation of symmetry relations between observables and initial conditions.

While the theoretical background for dual kinetic curves have been developed, experimental realizations often require further modifications of measurement techniques adapted for measurement of dual kinetic trajectories (of often concentrations of different species) from symmetrical initial conditions. For example, invariant quantities have been found theoretically in pulse-response “diffusion-reaction” systems (Temporal Analysis of Products, TAP) [9] and validated experimentally using as an example the water-gas shift catalytic reaction over iron catalyst [6].

In this paper, our objective is to develop dual kinetic experiments for invariant quantities in electrochemical reactions using a rotating ring-disk experimental setup. We perform numerical simulations in which we investigate the existence of dual kinetic trajectories from symmetrical initial conditions and the means by which time-invariant quantities can be extracted from concentration and current measurements. The theoretical predictions are validated in a rotating ring-disk electrode setup, where the kinetics of the reversible ferrocyanide-ferricyanide redox system is characterized with dual kinetic chronoamperometry. Finally, the technique is compared to other standard techniques for physical electrochemistry (e.g., cyclic voltammetry).

## Modeling results and discussion

### Model equations

The numerical simulations were performed with a generic reversible one-electron transfer Faradaic process:

$$A^{+}+e^{-}⇄B$$ (8)

An ordinary differential equation model is applied to approximate the kinetics of near surface concentrations of substances *A*^*+*^ and *B*. The mass balance equation considers kinetic contributions of the first order reactions with Butler-Volmer kinetics and mass transfer contributions using a Nernst diffusion layer (see S1 Appendix for derivation of the equations):

$$\frac{\text{d}c_{A}}{\text{d}t}=\frac{2}{a}(-k_{f}(V)c_{A}+k_{r}(V)c_{B})+\frac{2D_{A}}{a^{2}}(c_{A}^{0}-c_{A})$$ (9)

$$\frac{\text{d}c_{B}}{\text{d}t}=\frac{2}{a}(k_{f}(V)c_{A}-k_{r}(V)c_{B})+\frac{2D_{B}}{a^{2}}(c_{B}^{0}-c_{B})$$ (10)

where *t* is time, *a* is the diffusion layer thickness, *V* is the circuit potential, *c*_*A*_ and *c*_*B*_ are the near surface concentrations, *c*^*0*^_*A*_ and *c*^*0*^_*B*_ are the bulk concentrations, and *D*_*A*_ and *D*_*B*_ are the diffusion coefficients of substances *A*^*+*^ and *B*, respectively. The forward and backward rate constants depend on potential in an exponential form following Butler-Volmer kinetics

$$k_{f}(V)=k_{f}^{0}\;\exp\left[-(1-\beta )FV/RT\right]$$ (11)

and

$$k_{r}(V)=k_{r}^{0}\;\exp\left[\beta FV/RT\right]$$ (12)

where *β* is the transfer coefficient, *F* is the Faraday constant, *R* is the gas constant, *T* is temperature, and *k*_*f*_^*0*^ and *k*_*r*_^*0*^ are the rate constants measured at *V* = 0. (Positive potential is expected to drive anodic processes.) The generated current can be obtained from the overall rate of the reaction as

$$i(t)=AF(-k_{f}(V)c_{A}+k_{r}(V)c_{B})$$ (13)

where *A* is the surface area of the electrode; note that we consider the anodic current positive. (For simulation results we use electrode surface areas *A* = 1 cm^2^.) Slow linear sweep voltammograms are obtained by linear function of potential as a function of time:

$$V=V_{0}+vt$$ (14)

where *V*_0_ is the initial potential and *v* is the scan rate.

The Eqs 9–14 were numerically integrated with Gear’s method by using software XPPAUT[10].

### Simulation results

#### Linear sweep voltammetry

**Fig 1:**
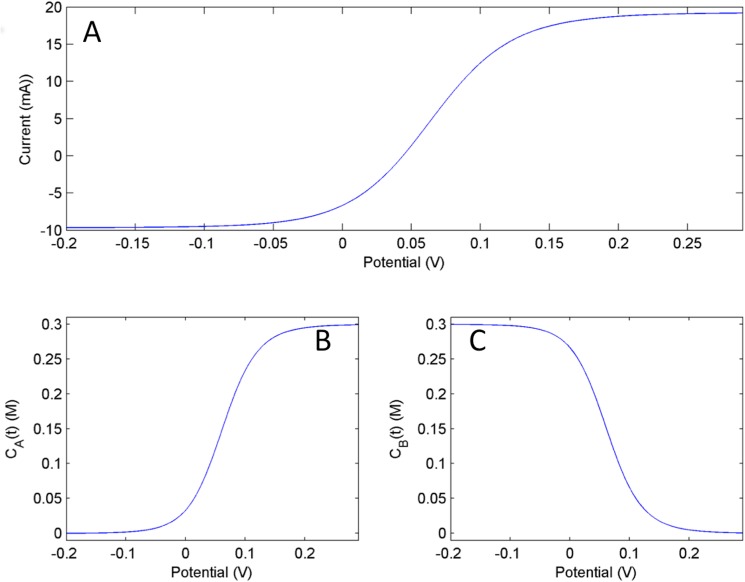
Numerical Simulation: Linear sweep voltammetry of model Eqs 9 and 10 A) current vs. time B) c_A_ vs. potential C) c_B_ vs. potential. Scan rate = 0.5mV/s.

Fig 1A shows results of numerical simulations of cathodic and an anodic linear sweep voltammetry (LSV). In the model consisting of Eqs 9–14, a very slow circuit potential scan (0.5mV/s) is performed that approximates the quasi-stationary voltammetric response at each potential. For the simulations, *a* = 0.01cm, *D*_*A*_ = *D*_*B*_ = 1x10^-5^cm^2^/s, *β* = 0.5, *k*_*f*_^*0*^ = 0.01cm/s, *k*_*r*_^*0*^ = 0.001cm/s, *c*^*0*^_*A*_ = 0.1M and *c*^*0*^_*B*_ = 0.2M were used as representative parameters. At the given bulk concentration, the open circuit potential (OCP) is observed at *V* = 0.041 V, where the current is zero. This potential corresponds to the thermodynamic equilibrium state. The voltammogram exhibits the expected sigmoidal shape, which is characterized by a sharp increase of the current magnitude for small overpotentials around the OCP and a current plateau for large overpotentials.

The anodic and cathodic limiting currents approach the expected values [11] *i*_lim,a_ = *FD*_*A*_*c*^*0*^_*B*_/*a* = 19.3mA and *i*_lim,c_ = -*FD*_*B*_*c*^*0*^_*A*_/*a* = -9.65mA; these values are well approximated (within 99.7%) at *V*_lim,a_ = 0.3 V and *V*_lim,c_ = -0.2 V. Fig 1B and 1C show the near surface concentrations of *A*^*+*^ and *B* as functions of the potential during the LSV. As expected, at the thermodynamic equilibrium point (at OCP) *c*_*A*_ = *c*^*0*^_*A*_, and *c*_*B*_ = *c*^*0*^_*B*_. In the anodic mass transfer limited region, all near surface substances are oxidized, therefore *c*_*A*_ = *c*^*0*^_*A*_+*c*^*0*^_*B*_ = 0.3 M, and *c*_*B*_ = 0; similarly, in the cathodic region with mass transfer limitation all species are present in the reduced form, *c*_*A*_ = 0, and *c*_*B*_ = *c*^*0*^_*A*_+*c*^*0*^_*B*_ = 0.3 M. Between these extreme values, the concentration vs. potential profile follows the expected sigmoid shape [12].

#### Dual kinetic curves

**Fig 2:**
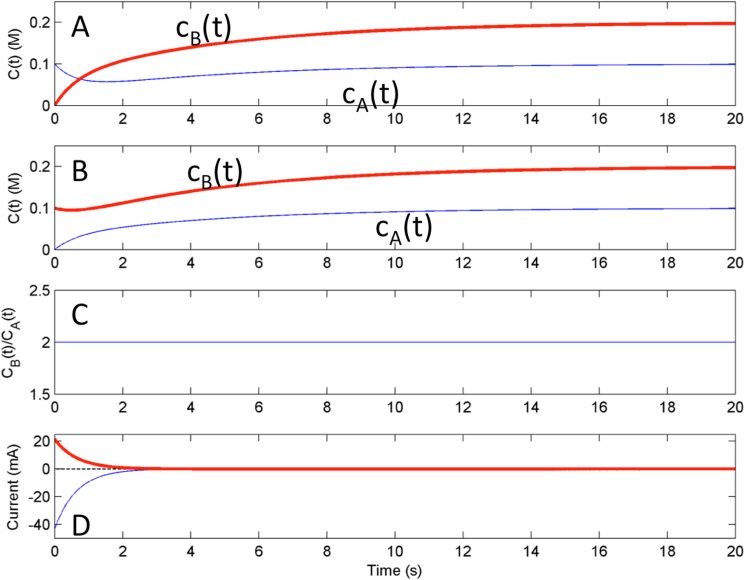
Numerical Simulation: Behavior at open circuit potential from different initial conditions. A) [c_A_(0),c_B_(0)] = [0.1M, 0M]. B) [c_A_(0),c_B_(0)] = [0M, 0.1M]. C) Ratio of concentrations obtained by dividing c_B_(t) from panel A by c_A_(t) from panel B D) current vs. time for initial conditions *i*’ [c_A_(0),c_B_(0)] = [0.1M, 0M] (bold), *i*” [c_A_(0),c_B_(0)] = [0M,0.1M] (thin), and scaled sum of the currents *i*’/2 + *i*” (dashed).

The presented model is used for simulating the dual kinetic trajectories that start from symmetric initial conditions. Fig 2A and 2B show the near surface concentration of *A*^*+*^ and *B* as functions of time for a system with following initial conditions *c*_*A*_(0) = 0.1 M, *c*_*B*_(0) = 0, and *c*_*A*_(0) = 0, *c*_*B*_(0) = 0.1 M, respectively. In the simulation the electrode potential *V* = 0.041 V is set to the OCP corresponding to bulk concentrations. The initial surface concentrations differ from bulk concentrations, therefore, there is a relaxation process to the thermodynamic equilibrium state; after the state is established, the surface concentrations reach the bulk ones. *c*_*A*_(*t*) (Fig 2A) and *c*_*B*_(*t*) (Fig 2B) are not monotonic, and a simple correlation between them is not apparent. However, *c*_*B*_(*t*) in Fig 2A and *c*_*A*_(*t*) in Fig 2B have a simple, monotonic relaxation to the corresponding bulk concentration values. The strong dependency between the variation of these concentration is shown in Fig 2C, where the ratio of concentrations *c*_*B*_(t) from Fig 2A and *c*_*B*_(t) form Fig 2B is shown as a function of time. In the limit as time goes to infinity, it expected that the concentration ratio will approach the ratio of bulk concentrations, which is 2. However, the figure shows that the concentration ratio is an invariant quantity at any time *t*> 0, i.e.,

$$\frac{c_{B}(t)}{c_{A}(t)}=\frac{\lim_{t\to \infty }c_{B}(t)}{\lim_{t\to \infty }c_{A}(t)}=\frac{c_{B}^{0}}{c_{A}^{0}}=K,\;\text{at any time}\;t>0$$ (15)

At potential corresponding to the OCP, the ratio of concentrations converges to an invariant quantity, which formally represents the equilibrium constant and can be calculated as the reaction quotient of bulk concentrations. Moreover, in accordance with Eq (15), the ratio of concentrations of the species (*c*_*B*_(*t*)/*c*_*A*_(*t*) obtained from symmetrical initial conditions is equal to the same invariant quantity (reaction quotient of bulk concentrations) at any time of this dual experiment.

Similar correlations are observed between the currents during the relaxation process (Fig 2D). A magnitude of the cathodic current generated from Fig 2B is exactly twice of the magnitude of the anodic current generated from Fig 2A for any time *t*>0. This implies that the relationship that was observed for the concentrations in Eq (15), also holds for the current, which is proportional for the rate of concentration variations in the system:

$$\frac{\left|i^{\prime }(t)\right|}{\left|i^{\prime \prime }(t)\right|}=\frac{c_{B}^{0}}{c_{A}^{0}}=K$$ (16)

where *i*’(*t*) and *i*”(t) are the current obtained from symmetrical initial conditions with *c*_*B*_(0) = 0 and *c*_*A*_(0) = 0, respectively. A simple test for the relationship in Eq (16) can be done by plotting the quantity related to *i*’(*t*)*c*^*0*^_*A*_ +*i*”(*t*)*c*^*0*^_*B*_ as a function of time; when the relationship holds we should get a zero normalized current for the any time *t*> 0, as shown by the dashed line in Fig 2D.

#### Dependence dual kinetic trajectories on bulk concentration and initial conditions

**Fig 3:**
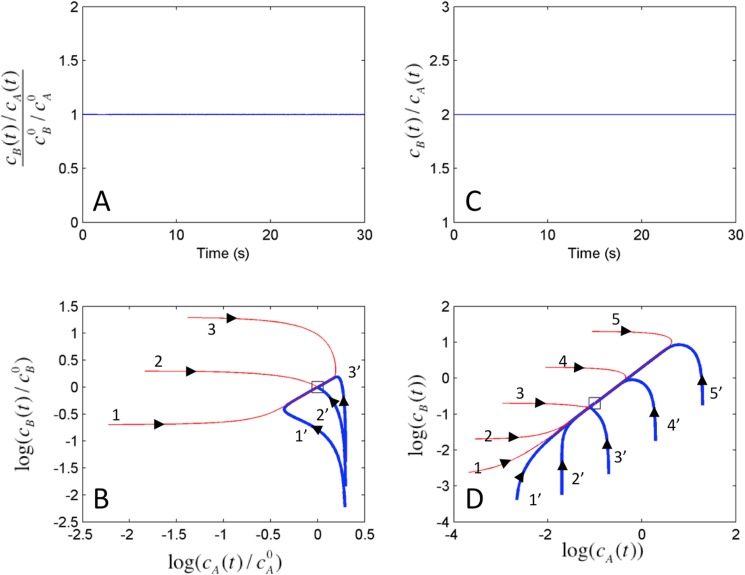
Numerical Simulation: Test of reciprocal relationship for different bulk concentrations (left side of panel) and different initial conditions (right side of the panel). A) The quotient of reciprocal trajectories *c*_B_(*t*)/*c*_A_(*t*) for different bulk concentrations divided by their bulk concentration ratio ($c_{B}^{0}/c_{A}^{0}$ = 0.1,1, and 10. $c_{A}^{0}$ was kept constant at 0.1M; only $c_{B}^{b}$ was changed). Initial conditions: [c_A_(0),c_B_(0)]_1_ = [0.2 M, 0] for *c*_B_(t) and [*c*_A_(0),*c*_B_(0)]_2_ = [0,0.2 M] for *c*_A_(*t*); *c*_B_(*t*) from condition 1 is divided by *c*_A_(*t*) from condition 2 to get the quotient. B) Phase space trajectories corresponding to results in panel A. The initial condition for trajectories 1,2, and 3 is [c_A_(0),c_B_(0)]_1_ = [0,0.2 M]. For trajectories 1’, 2’, and 3’ the initial condition is [c_A_(0),c_B_(0)]_2_ = [0.2 M, 0]. The bulk concentration ratios: for (1 and 1’), (2 and 2’) and (3 and 3’) are 0.1 M, 1 M, and 10 M, respectively. C) Ratio *c*_B_(*t*)/*c*_A_(*t*) for different initial conditions in the form [c_A_(0),c_B_(0)] = [x,0] for determining *c*_B_(*t*) and [0,x] for *c*_A_(*t*) (x = 0.002 M, 0.02 M, 0.2 M, 2 M, 20 M). The bulk ratio was kept constant at $c_{B}^{0}/c_{A}^{0}$ = 2. D) Phase state trajectories corresponding to results in panel C. The initial conditions for trajectories 1, 2, 3, 4, and 5 follow the same form as panel C with [*c*_A_(0),*c*_B_(0)]_1_ = [0,x] where x = 0.002 M, 0.02 M, 0.2 M, 2 M, 20 M respectively. For trajectories 1’, 2’, 3’, 4’, and 5’ the initial conditions are [c_A_(0),c_B_(0)]_1_ = [x,0] where x = 0.002 M, 0.02 M, 0.2 M, 2 M, 20 M respectively.

The invariance of the ratio of concentrations in Eq (15) was numerically tested for different bulk concentrations (Fig 3A and 3B), and different initial conditions (Fig 3C and 3D). The dual kinetic trajectories are shown in Fig 3B for three different ratios (*c*^*0*^_*B*_/ *c*^*0*^_*A*_ = 0.1 M, 1M, and 10M) starting from symmetrical initial conditions [*c*_*B*_(0), *c*_*A*_(0)] = [0,0.2M] and [0.2M, 0]. (Note that for each of these curves a different circuit potential was used that corresponds to the OCP of the reaction system). Although the kinetic trajectories show diverse routes, for any time *t*> 0 the ratio of *c*_*B*_(*t*) and *c*_*A*_(*t*) obtained from the corresponding kinetic trajectories (where *c*_*B*_(0) and *c*_*A*_(0) is zero, respectively) is equal to the ratio of bulk concentrations (see Fig 3A). Similarly, simulations were done with different initial conditions and the same bulk concentrations *c*^*0*^_*A*_ = 0.1M, *c*^*0*^_*B*_ = 0.2 M. The initial conditions for 5 dual kinetic trajectories were set to [*c*_*A*_(0),*c*_*B*_(0)] = [x,0] and [*c*_*A*_(0),*c*_*B*_(0)] = [0,x] with x = 0.002 M, 0.02 M, 0.2 M, 2 M, 20M. As it can be seen from Fig 3D, all kinetic trajectories tend to the same equilibrium state corresponding to the ratio of the bulk concentrations; the trajectories also exhibit a relatively fast initial period where large changes occur followed by a relatively slow variation along the diagonal of the of the graph. The presence of such manifold is a characteristic nature of reactions systems with time-scale separation; in this example the reaction timescale (related to the inverse of rate constant) is smaller than the diffusion timescale (proportional to the inverse of the diffusion constant). Nonetheless, as it is shown in Fig 3C, the ratio *c*_*B*_(t)/*c*_*A*_(t) was equal to 2 (*c*^*0*^_*B*_/*c*^*0*^_*A*_) for each pair of the corresponding dual kinetic trajectories for *t*>0.

#### Generation of initial conditions with pre-polarization

Results shown in Figs 2 and 3 indicate that the ratio of concentrations extracted from dual kinetic measurements at OCP (equilibrium potential) are invariant quantities and equal to the reaction quotient obtained from the bulk concentrations. The dual kinetic trajectories require symmetrical initial conditions, which might not be easy to establish in an actual experimental implementation. Here we consider a relatively simple method for the initiation of the reactions from symmetrical initial conditions. Fig 1 shows that at large anodic overpotential almost all of the substances are oxidized, therefore *c*_*B*_ = 0, *c*_*A*_ = *c*^*0*^_*A*_+*c*^*0*^_*B*_. Similarly, at large cathodic overpotential the oxidized form of the species are fully reduced, i.e., *c*_*B*_ = *c*^*0*^_*A*_+*c*^*0*^_*B*_ and *c*_*A*_ = 0. We thus see that conditions obtained at large cathodic/ anodic overpotentials can serve as symmetrical initial conditions required for measurement of the dual kinetic trajectories.

**Fig 4:**
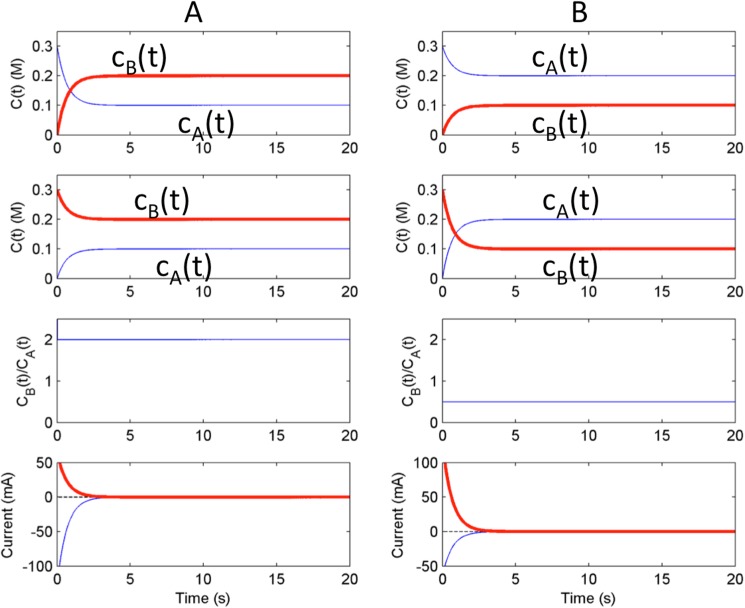
Numerical Simulation: Test of reciprocal relationships from initial conditions obtained with cathodic and anodic mass transfer limited pre-polarizations. A: bulk concentration ratio $c_{B}^{0}/c_{A}^{0}$ = 2. B) bulk concentration ratio $c_{B}^{0}/c_{A}^{0}$ is 0.5. The top row of figures shows behavior at open circuit potential from anodic pre-prepolarization conditions (*V* = +0.5 V). The second row shows behavior at open circuit potential from cathodic pre-polarization (*V* = -0.5 V). The third row shows the ratio of concentrations obtained by dividing *c*_B_(*t*) from the first row figure by *c*_A_(*t*) from the corresponding second row figure. The bottom row of figures show the currents for initial conditions *i*_A_[*V* = +0.5 V] (bold),i_C_[*V* = -0.5 V] (solid) and the scaled sum of the currents (dashed). For the left figure, the scaled sum is *i*_A_/2 + *i*_C_. For the right figure, the scaled sum is *i*_A_ + *i*_C_/2.

Fig 4 A shows the features of the dual kinetic trajectories obtained with *c*^*0*^_*A*_ = 0.1M and *c*^*0*^_*B*_ = 0.2 M starting from initial conditions that was obtained by setting circuit potentials to the anodic mass transfer limit (*V* = *V*_*lim*,*a*_ = 0.5V, top row) or to the cathodic mass transfer limit (limit (*V* = *V*_*lim*,*c*_ = -0.5V, second row). With these initial conditions the concentration vs time plots exhibit a monotonic relaxation to the equilibrium state. As expected, the ratio of concentrations *c*_*B*_(*t*) and *c*_*A*_(*t*) obtained from the anodic and cathodic transient is *c*^*0*^_*B*_/*c*^*0*^_*A*_ = 2 constant value for *t*> 0 (see Fig 3, third row). The ratio of the magnitudes of the cathodic and anodic relaxation currents are also equal to *c*^*0*^_*B*_*/c*^*0*^_*A*_ = 2 (see Fig 4, bottom panel). Similarly, when the bulk concentrations of the substances are switched (Fig 4B, *c*^*0*^_*A*_ = 0.2 M, *c*^*0*^_*B*_ = 0.1 M), the *c*_*B*_(*t*) /*c*_*A*_(*t*) obtained from the dual kinetic trajectory changed to *c*^*0*^_*B*_/*c*^*0*^_*A*_ = 0.5, and now the magnitude of the cathodic relaxation currents are half of the anodic relaxation currents.

The numerical simulations thus confirm that dual kinetic trajectories exhibit invariant features characteristic of first-order reversible reactions. With a disk electrode, a convenient way of generating of two symmetrical initial conditions is to pre-polarize the electrode at high anodic and cathodic potentials where the mass-transfer limited reactions fully consume one of the reactants.

## Experimental results and discussion

We designed a series of experiments in which the theoretically developed technique for dual kinetic chronoamperometry can be validated in a single electron transfer reversible reaction, the oxidation of ferrocyanide to ferricyanide ions. The goal is to show the existence of time-invariant ratio of concentrations of the oxidized and reduced species (Eq 14) and the corresponding currents (Eq 15) in dual kinetic experiments that start with symmetrical near-surface concentrations at OCP conditions.

### Experimental procedures

#### Experimental Setup

A standard electrochemical setup with the rotating Pt disk–Au ring working electrodes (Pine Instruments AFMSRCE), Hg/Hg_2_SO_4_/saturated K_2_SO_4_ reference electrode, and a Pt wire counter electrode was used. (All potentials are given with respect to the reference electrode.) The electrolyte consisted of a mixture of given concentrations of ferrocyanide (Sigma 98.5% ReagentPlus), ferricyanide (Acros Organics 99%+ for analysis), and 1 M potassium nitrate (Acros Organics99%+) background electrolyte. The 100 mL of the solution was used in a water-jacketed reactor with temperature controlled at 25°C. Nitrogen gas was bubbled through the solution for 15 minutes and stopped prior to the start of experimentation. Pine AfterMath software was used to control the bipotentiostat (Pine model AFCBP1).

#### Data collection

The experiment is a traditional potential step chronoamperometric measurement. Before each experiment the open circuit potential (OCP) was measured. This potential value was used during the experiment after the pre-polarization. At OCP, the surface concentrations of ferro/ferricyanide ions are considered to be equal to the solution bulk concentrations. After the OCP determination, two slow (*v* = 0.5mv/s) linear sweep voltammetry scans were performed. In the first sweep, the potential was scanned to anodically (increasing potential) from the OCP to determine the potential value (*V*_*lim*,*a*_) at which the anodic mass transfer limiting current is reached. In the second experiment, a similar scan was performed in the cathodic direction to determine the potential value (*V*_*lim*,*c*_) at which the cathodic mass transfer limiting current is obtained.

The ring electrode was set to a constant potential *V*_*ring*_ for the entire experiment; the potential was set to *V*_*lim*,*a*_ for detection of ferrocyanide, and to *V*_*lim*,*c*_ for detection of the ferricyanide ions. In the anodic pre-polarization experiments, the potential of the disk electrode was set to *V*_*lim*,*a*_ for 10 seconds after which the potential was switched to the OCP, and the currents of both the disk and ring electrodes were recorded for a duration of 0.6 s during which the disk electrode reached the equilibrium state with the zero current. The cathodic pre-polarization experiments were performed similarly but the circuit potential of the disk electrode was set to *V*_*lim*,*c*_ before the switch of the potential to OCP.

#### Calculation of near surface concentrations from ring current measurements

The near surface concentrations of the reduced and oxidized species are determined with standard shielding experiments from the ring currents. [13, 14] The ring currents measured from each shielding experiments, in which the ring oxidizes/reduces products created at the disk that diffuse outward, are directly proportional to concentrations at the ring [13, 14]. When the disk is at the OCP, the concentration of electroactive species is the same as the bulk concentration. When the disk is polarized at the cathodic or anodic mass transfer region, the ring current measures the bulk concentration which is modified by the known concentration contribution from the disk. Therefore, the ring current measurements with disk potential set at the OCP and at the mass transfer limited region present two data points with known concentrations and ring currents. These two points are used to generate a calibration line for calculations the near surface concentrations of the disk from the ring current at any point during the transients.

Equations which are used for the calculation of ferro- or ferricyanide concentrations near the disk surface depend on the type of pre-polarizations (anodic or cathodic).

### Anodic transients

For detection of the ferricyanide concentration (*A*^*+*^), the ring potential is set to *V*_*lim*,*a*_ and

$$c_{A}(t)=c_{A}^{0}+\frac{\left|i_{r}(t)-i_{r}(OCP,a)\right|}{\left|i_{r}(lim,a)-i_{r}(OCP,a)\right|}c_{B}^{0},$$ (17)

where *i*_r_(*t*) is the ring current measured during the transient, and *i*_r_(*OCP*,*a*) and *i*_r_(*lim*,*a*) are the ring currents obtained at the OCP and at the limiting anodic potential, respectively. Similarly, for detection of the ferrocyanide concentration (*B*), the ring potential is set to *V*_*lim*,*c*_ and

$$c_{B}(t)=\frac{\left|i_{r}(t)-i_{r}(OCP,c)\right|}{\left|i_{r}(lim,c)-i_{r}(OCP,c)\right|}c_{B}^{0},$$ (18)

where *i*_r_(*OCP*,*c*) and *i*_r_(*lim*,*c*) are the ring currents obtained at the OCP and at the limiting cathodic potential, respectively. In accordance with presented equations, two experiments were required for measurements of the surface concentration transients of *A*^*+*^ and *B*. In these experiments, the ring electrodes are polarized anodically and cathodically, respectively.

### Cathodic transients

For detection of concentration of the ferricyanide (*A*), the ring potential is set to *V*_*lim*,*a*_ and

$$c_{A}(t)=\frac{\left|i_{r}(t)-i_{r}(OCP,a)\right|}{\left|i_{r}(lim,a)-i_{r}(OCP,a)\right|}c_{A}^{0}$$ (19)

Similarly, for detection of ferrocyanide concentration (*B*), the ring potential is set to *V*_*lim*,*c*_ and

$$c_{B}(t)=c_{B}^{0}+\frac{\left|i_{r}(t)-i_{r}(OCP,c)\right|}{\left|i_{r}(lim,c)-i_{r}(OCP,c)\right|}c_{A}^{0}$$ (20)

### Linear sweep voltammetry

**Fig 5:**
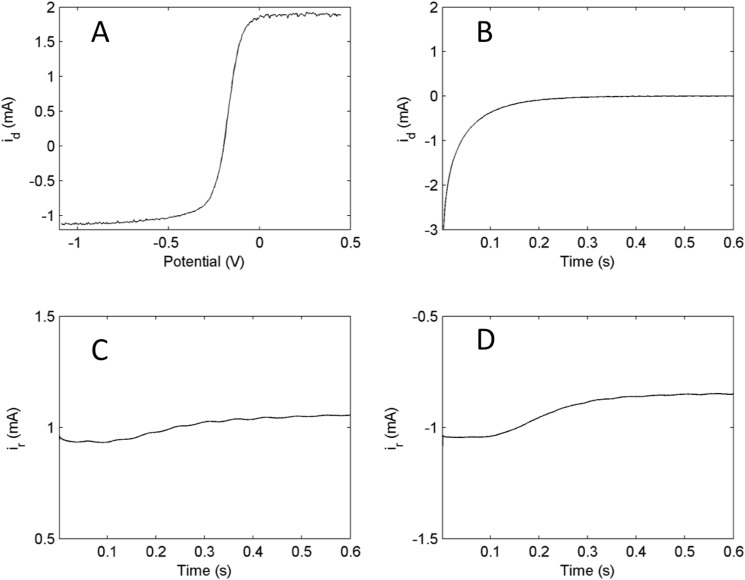
Experiments: Primary data for linear sweep voltammetry and representative kinetic curves at open circuit potential. A) Linear sweep voltammetry curve (scan rate = 10mV/s) of the system B) Disk current relaxation when disk potential is changed from cathodic pre-polarization (*V* = -1000mV) to the open circuit potential (*V* = -197mV) C) Ring current relaxation when ring current is held at the anodic limiting potential (*V* = 200mV) while disk potential is changed as in panel B. D) Ring current relaxation when ring current is held at the cathodic limiting potential (*V* = -1000mV) while disk potential is changed as in panel B. Rotation rate = 1000rpm, *T* = 25°C, $c_{Fe3+}^{0}$ = 0.01M, $c_{Fe2+}^{0}$ = 0.02M.

We used ferricyanide as the oxidized species (component A) of the solution and ferrocyanide as the reduced species (component B). The experiments were done using a rotating ring-disk electrode under the following conditions: rotation rate = 1000rpm, *T* = 25°C, $c_{Fe3+}^{0}$ = 0.01M, $c_{Fe2+}^{0}$ = 0.02M. Fig 5 shows the primary experimental data collected during a linear sweep voltammetry experiment with the ring-disk electrode. Fig 5A shows the linear sweep scan in both the anodic and cathodic directions. The current at the anodic limiting region is 1.83 mA and -1.02 mA at the cathodic region. The reason the absolute value of the cathodic current is not exactly half of the anodic limiting current is because the two species have slightly different diffusion coefficients (ferrocyanide = 6.0x10^-10^m^2^s^-1^ and ferricyanide = 7.0x10^-10^m^2^s^-1^)[15].

### Dual kinetic curves

Fig 5B gives a typical example of the disk current in an experiment with cathodic pre-polarization. The current relaxes quickly (in about 0.3 s) to 0 when the potential is set to OCP. Fig 5C and 5D show the corresponding ring currents that are associated with the disk current change where the ring electrode was held at the anodic and cathodic limiting potentials, respectively. The corresponding concentrations of the oxidized and reduced species can be calculated using Eqs (19) and (20).

**Fig 6:**
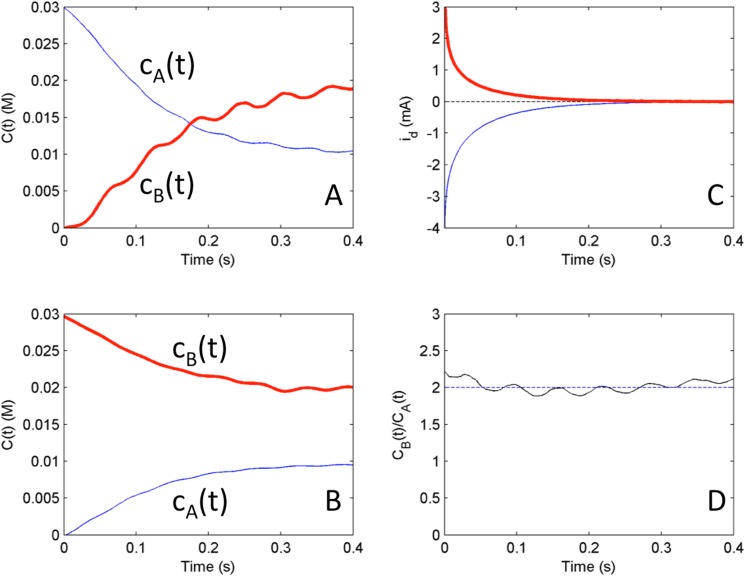
Experiments: Test of reciprocal relationships from initial conditions of cathodic and anodic mass transfer limited pre-polarizations for system where the bulk concentration ratio $c_{B}^{0}/c_{A}^{0}$ = 2. A) Relaxation of concentrations from initial condition corresponding to anodic pre-polarization (*V* = 200mV) at equilibrium potential (OCP = -197mV) B) Relaxation of concentrations from initial condition corresponding to cathodic pre-polarization (*V* = -1000mV) at equilibrium potential (OCP = -197mV) C) disk currents from both anodic (bold) and cathodic (solid) initial conditions and their scaled sum i_A_/2 + i_C_ (dashed line) D) ratio of concentrations c_B_(t)/c_A_(t) with a dashed line representing the bulk concentration ratio $c_{B}^{0}/c_{A}^{0}$ = 2.

We have performed a set of dual kinetic experiments with *c*_*A*_^*0*^ = 0.01 M and *c*_*B*_^*0*^ = 0.02 M and the results are shown in Fig 6. In the first of the dual kinetic experiments (Fig 6A) the disk electrode was anodically pre-polarized (*V* = 200 mV), and the potential was set to OCP (-197 mV). The corresponding disk current (see Fig 6C) was recorded, and the ring currents were used to calculate the near surface concentrations in Fig 6A. As expected, the initial concentrations are 0.03M for ferricyanide and 0 for ferrocyanide. During the course of the experiment the concentrations relax to the bulk concentration with a single crossing point. (The 16.6 Hz fluctuation seen in the curves is due to the graphite brush connection inside the rotating electrode. These fluctuations can be seen to different extent in all the experiments.)

In the second dual kinetic experiment, the electrode was pre-polarized cathodically (*V* = -1000 mV) and the corresponding disk currents and the near surface concentrations were determined at the OCP (see Fig 6C and 6B, respectively). The initial concentrations are now 0 for ferricyanide and 0.03 M for ferrocyanide. During the experiment the concentration relax again to the bulk concentration, but this time without a crossing point.

Now the dual kinetic curves can be analyzed by plotting the experimental determined ratio *c*_*B*_(t)/*c*_*A*_(t) from the two experiments (see Fig 6D). The ratio fluctuates around the expected bulk concentration ratio $c_{B}^{0}/c_{A}^{0}$ = 2. Therefore, we see the existence of the invariant quantity predicted by the numerical simulations. While the concentration measurements using the ring electrodes could have some confounding factors, the presence of invariant quantities can be clearly seen in comparison of the disk currents in Fig 6C. The dashed line in the plot *i*_A_(*t*)/2 + *i*_C_(*t*) is equal to nearly zero (-0.012mA) as predicted by the numerical simulations shown in Fig 4A.

**Fig 7:**
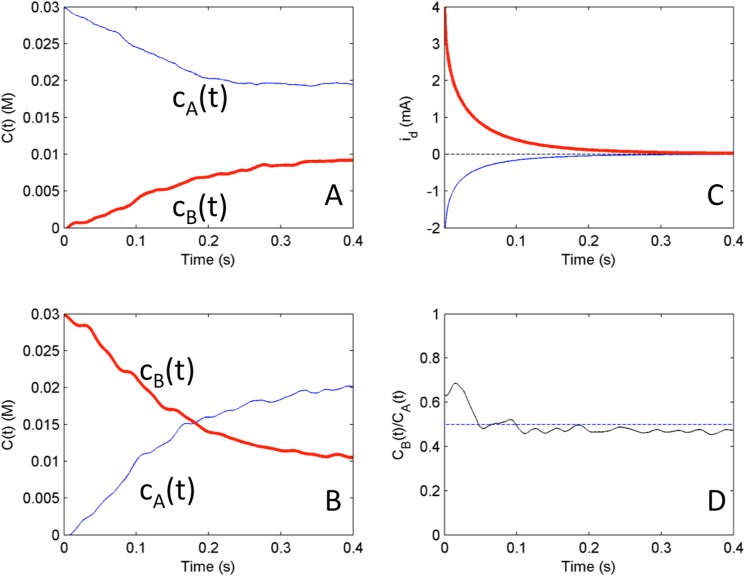
Experiments: Test of reciprocal relationships from initial conditions of cathodic and anodic mass transfer limited pre-polarizations for system where the bulk concentration ratio $c_{B}^{0}/c_{A}^{0}$ = 0.5. A) Relaxation of concentrations from initial condition corresponding to anodic pre-polarizatin (V = 100mV) at equilibrium potential (OCP = -162mV) B) Relaxation of concentrations from initial condition corresponding to cathodic pre-polarization (V = -500mV) at equilibrium potential (OCP = -162mV) C) disk currents from both anodic (bold) and cathodic (solid) initial conditions and their scaled sum i_C_/2 + i_A_ (dashed line) D) ratio of concentrations c_B_(t)/c_A_(t) with a dashed line representing the bulk ratio $c_{B}^{0}/c_{A}^{0}$ = 0.5.

Similar dual-kinetic experiments were also performed with $c_{B}^{0}/c_{A}^{0}$ = 0.5 instead of 2; the results are summarized in Fig 7. In the first dual kinetic experiment (Fig 7A) with anodic pre-polarization (with initial conditions 0.03M for ferricyanide and 0 M for ferrocyanide) the relaxation to equilibrium point is now without crossing point. In the second dual kinetic experiment (Fig 7B) with cathodic pre-polarization (with initial conditions 0 M for ferricyanide and 0.03 M for ferrocyanide) the relaxation to equilibrium point is through a single crossing point. In the *c*_B_(*t*)/*c*_A_(*t*) plot we again observe a near time invariant ratio (close to $c_{B}^{0}/c_{A}^{0}$ = 0.5) with slightly larger values in the beginning, and slightly smaller (0.47) value at the end. For the deviation at the beginning, we often observed a slight delay (order of ms) in the increase of ring current; such delay can affect the concentration ratio at beginning where small change can result in large change in the ratio. Nonetheless, the disk currents in Fig 7C scale according to expected ratio, therefore, the deviation in Fig 7D are most likely related to small errors in the conversion of ring currents to concentrations. The experiments further confirm the use of the dual kinetic chronoamperometry and support the numerical simulation results shown in Fig 4.

## Conclusions

In conclusion, reciprocal relations between the kinetic curves provide a unique possibility to extract the non-steady state trajectory starting from one initial condition based only on the equilibrium constant and the trajectory which starts from the symmetrical initial condition. Dual kinetic chronoamperometry is proposed as a novel technique for exploration of kinetic features of electrochemical reactions. Kinetic information is extracted from two experiments: each experiment consisted of setting the disk electrode to an equivalent far-from-equilibrium potential, such as the anodic or cathodic limit, and allowing each to relax to equilibrium defined by the Nernst potential. Numerical simulations indicate that the proper ratio of the transient kinetic curves obtained from cathodic and anodic mass transfer limited regions give thermodynamic time invariances related to the reaction quotient of the bulk concentrations. Experimental tests with the ferrocyanide/ferricyanide system further confirm the principle: the concentrations of the oxidized and reduced species followed reciprocal paths as they relaxed toward equilibrium as long as both started from an equivalent state.

In addition, dual kinetic chronoamperometry can be used to test the reversibility of a system. A system can have a reversibility factor ranging from total irreversibility to Nernstian reversibility depending on the relationship between the forward and reverse reaction rate constants[16]. Ideally the system needs to be as close to Nernstian reversibility as possible to ensure that an electrochemical inactive species is not inhibiting either trajectory. As a result of this need for reversibility, the experiment can also provide an alternative method to test for reversibility besides cyclic voltammetry. If the two species have reciprocal kinetic curves, then the system must also be reversible. This method provides a fundamentally potentiostatic experimental avenue to discover reversibility in a system. (The potential step in our methodology was required for generation of initial conditions; if these conditions are generated with different methods, e.g., with a flow cell, a fully potentistatic method is feasible.) At the present stage of development, the technique was demonstrated for a fixed diffusion layer thickness and for similar values of diffusion coefficients for the electroactive species. Future work could include extension of the method for more complex reaction mechanisms, far-from-equilibrium states (e.g., disk potentials away from the Nernst potentials), and for standing electrodes with varying diffusional layer thickness. In particular, applications to battery systems relating charging and discharging processes are promising as in these processes the initial conditions can be fundamentally symmetrically related (e.g., charged or discharged cell).

## Supporting information

S1 Appendix Derivation of the model Eqs (9) and (10). (PDF)
