# Supplementary material for: Dual kinetic curves in reversible electrochemical systems
Source: PLoS One. 2017 Mar 30;12(3):e0173786. doi: 10.1371/journal.pone.0173786 (PMC5373521; doi:10.1371/journal.pone.0173786)
Supplement: S1 Appendix — (PDF) [file pone.0173786.s001.pdf]

S1 Appendix

Dual kinetic curves in reversible electrochemical systems

Michael J. Hankins[1], Gregory S. Yablonsky[2], and István Z. Kiss[1],

[1]Department of Chemistry, Saint Louis University, 3501 Laclede Av., St. Louis, MO 63103

[2]Parks College of Engineering, Aviation and Technology, Saint Louis University, 3450, Lindell Blvd, St. Louis, MO 63450

Model equations

Mass balance equation is formulated for a reversible first order electrochemical reactions with mass transfer described through a Nernst diffusion layer approach [RA1]. The rate law of the reaction

$$A^{+} + e^{-} \leftrightharpoons B \qquad \text{(A1)}$$

follows first order kinetics with the following forward (r_f) and reverse (r_r) rates:

$$r_f = k_f c_A \qquad \text{(A2)}$$

$$r_r = k_r c_B \qquad \text{(A3)}$$

where c_A and c_B are the surface concentrations of the species A^+ and B, respectively, and k_f and k_r are the first order forward and reverse rate constants.

We assume that the concentration layer profile changes linearly from the surface concentrations (c_A and c_B) to the bulk concentrations (c^0_A and c^0_B) within a diffusion layer with thickness a.

When the surface concentration changes within an infinitesimally small time d*t* for time for species J, the amount of change in the electroactive species is

$$\frac{1}{2}\big(c_J(t+dt)-c_J(t)\big)a \qquad \text{(A4)}$$

In the same time interval, this amount should be equal to the amount of species diffused and reacted, which can be calculated from Fick's first law and the rate law for the reactions, respectively:

$$\frac{D(c_J^0-c_J)}{a}+\sum_R \nu_J r_R \qquad \text{(A5)}$$

where D is diffusion constant and ν_J are the stoichiometric numbers for species J in reactions *R* with rate r_R. Equations A4 and A5 must be equal, therefore

$$\frac{dc_J}{dt}=\frac{2}{a}\left(\sum_R \nu_J r_R+\frac{D(c_J^0-c_J)}{a}\right) \qquad \text{(A6)}$$

For species A^+ and B

$$\frac{dc_A}{dt}=\frac{2}{a}\left(-k_f c_A+k_r c_B+\frac{D(c_A^0-c_A)}{a}\right) \qquad \text{(A7)}$$

$$\frac{dc_B}{dt}=\frac{2}{a}\left(k_f c_A-k_r c_B+\frac{D(c_B^0-c_B)}{a}\right) \qquad \text{(A8)}$$

We use these equations in the manuscript to describe the behavior.

References

RA1. Koper, MTM, Sluyters, JH. Electrochemical oscillators: their description through a mathematical model. J Electroanal Chem. 1991;303:73-94.
